# Targeting Glutamine Metabolism in Prostate Cancer

**DOI:** 10.31083/j.fbe1501002

**Published:** 2023-01-04

**Authors:** Neil Bhowmick, Edwin Posadas, Leigh Ellis, Stephen J Freedland, Dolores Di Vizio, Michael R Freeman, Dan Theodorescu, Robert Figlin, Jun Gong

**Affiliations:** 1 Department of Medicine, Division of Hematology and Oncology, Samuel Oschin Comprehensive Cancer Institute, Cedars-Sinai Medical Center, Los Angeles, CA 90048, USA; 2 Department of Surgery, Division of Urology, Cedars-Sinai Medical Center, Los Angeles, CA 90048, USA; 3 Department of Surgery, Division of Cancer Biology and Therapeutics, Biomedical Sciences, and Pathology and Laboratory Medicine, Cedars-Sinai Medical Center, Los Angeles, CA 90048, USA

**Keywords:** glutamine, prostate cancer, glutaminase, MYC, androgen receptor, mTOR, castrate-resistance, PTEN

## Abstract

Glutamine is a conditionally essential amino acid important for cancer cell proliferation through intermediary metabolism leading to *de novo* synthesis of purine and pyrimidine nucleotides, hexosamine biosytnehsis, fatty acid synthesis through reductive carboxylation, maintenance of redox homeostasis, glutathione synthesis, production of non-essential amino acids, and mitochondrial oxidative phosphorylation. Prostate cancer has increasingly been characterized as a tumor type that is heavily dependent on glutamine for growth and survival. In this review, we highlight the preclinical evidence that supports a relationship between glutamine signaling and prostate cancer progression. We focus on the regulation of glutamine metabolism in prostate cancer through key pathways involving the androgen receptor pathway, *MYC*, and the PTEN/PI3K/mTOR pathway. We end with a discussion on considerations for translation of targeting glutamine metabolism as a therapeutic strategy to manage prostate cancer. Here, it is important to understand that the tumor microenvironment also plays a role in facilitating glutamine signaling and resultant prostate cancer growth. The druggability of prostate cancer glutamine metabolism is more readily achievable with our greater understanding of tumor metabolism and the advent of selective glutaminase inhibitors that have proven safe and tolerable in early-phase clinical trials.

## 1. Introduction

Prostate cancer is the most commonly diagnosed cancer and the second leading cause of cancer mortality in men with a projected 268,490 new cases and 34,500 deaths in 2022 [1]. Androgen targeted therapy (ATT) remains a cornerstone of treatment in almost all stages of prostate cancer given the near universal dependence on androgen receptor (AR) signaling for tumor growth and proliferation [2,3]. For example, androgen deprivation therapy (ADT) is used in the neoadjuvant and/or adjuvant treatment of localized prostate cancer, while ADT is incorporated into salvage therapies for local relapse. In systemic disease, ADT is often combined with chemotherapy or novel hormonal therapies (NHT) that inhibit the androgen signaling axis. ADT can also be combined with NHT in those with prostate cancer who have developed castration resistance but are non-metastatic (M0). Metastatic castrate-resistant prostate cancer (mCRPC) often represents the eventual development of resistance to therapies against the androgen pathway. Despite advancements in systemic therapies often added to a backbone of ADT in metastatic castrate-sensitive prostate cancer (mCSPC) and mCRPC, metastatic prostate cancer remains incurable and novel therapeutic strategies to improve patient outcomes are a high unmet need [4].

Greater understanding of tumor metabolism across multiple cancer models have shown that cancer cells are fundamentally reliant on glucose and glutamine to fuel anabolic processes necessary to sustain tumor proliferation [5,6]. Although long known to be less glycolytic (and hence less fluorodeoxyglucose or FDG avid), highly lipogenic, and reliant on oxidative phosphorylation than other solid tumor types, prostate cancer metabolism is heavily driven by AR signaling with increasing evidence of dependency on glutamine metabolism to support prostate cancer cell growth [5,6]. In fact, during neoplastic transformation, prostate cells undergo metabolic reprogramming to support tumor growth and proliferation involving pathways such as amino acid metabolism, lipid biosynthesis, and the tricarboxylic acid (TCA) cycle [7,8]. The purpose of this review is to highlight the latest understanding of the role of glutamine metabolism in supporting prostate cancer proliferation. More importantly, we review novel therapeutic strategies to target the glutamine dependency of prostate cancer in preclinical models. We end with a discussion on considerations for translation of this therapeutic approach in the clinical management of patients with prostate cancer.

## 2. Overview of Glutamine Metabolism

**Fig. 1.**
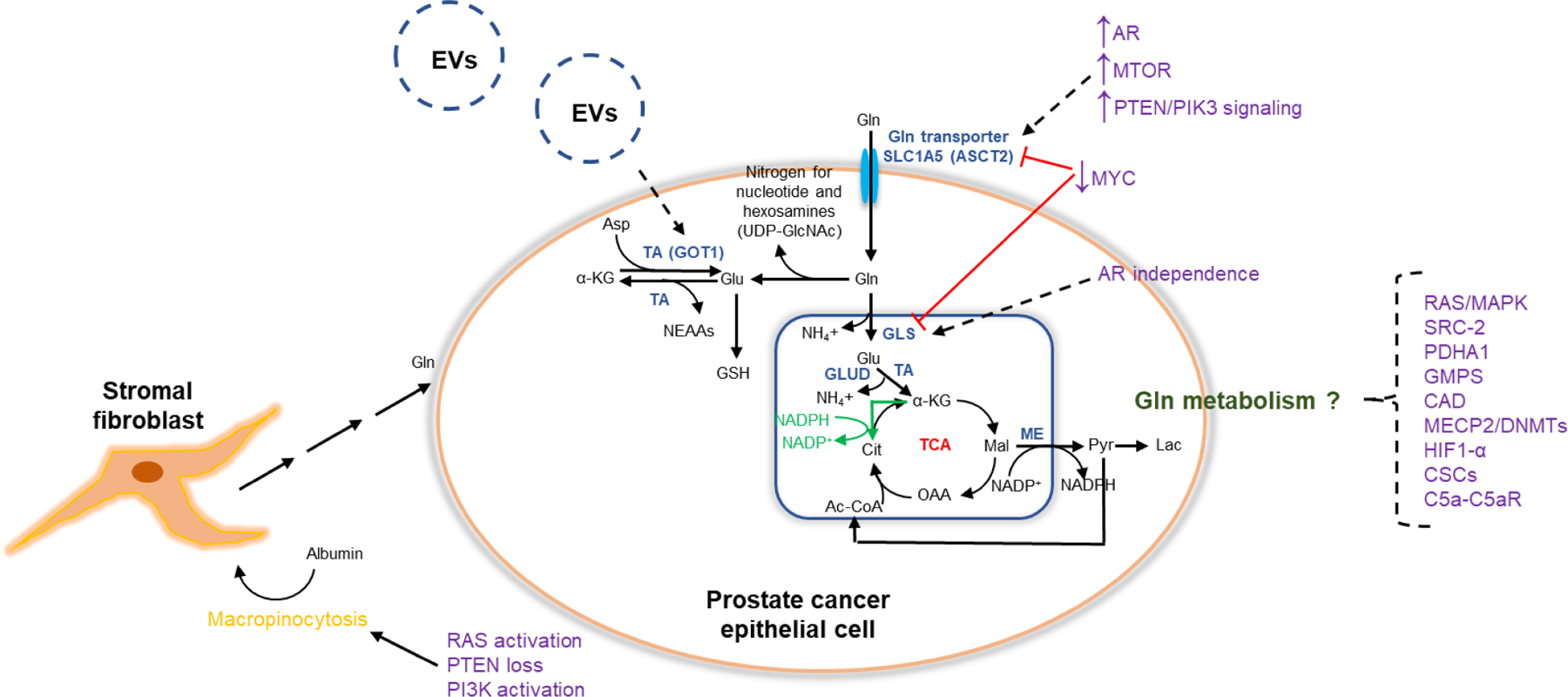
Glutamine metabolism and key regulators in prostate cancer. Glutamine supports prostate cancer cell growth through intermediary metabolism. Entry into the cell is facilitated by the principle membrane glutamine transporter (SLC1A5 or ASCT2). Glutamine contributes to *de novo* synthesis of both purine and pyrimidine nucleotides and UDP-GlcNAc as part of the hexosamine biosynthesis pathway. When converted to glutamate, glutamine contributes to GSH synthesis, production of NEAAs, and mitochondrial oxidative phosphorylation as glutamine is converted to glutamate by GLS with the latter converted to *α*-KG for entry into the TCA cycle to produce other intermediates and eventually NADH, FADH2, and ATP. The carbon skeleton of glutamine exits from the TCA cycle as malate with subsequent conversion to pyruvate and then acetyl-CoA for re-entry into the TCA cycle. Oxidation of glutamine also maintains redox homeostasis by reduction of NADP+ to NADPH by malic enzyme through the conversion of glutamine to pyruvate. Lastly, glutamine can also serve as an alternative carbon source to fuel fatty acid synthesis through reductive carboxylation where NADPH is consumed to produce citrate (green arrows). Key regulators of glutamine metabolism include the AR pathway, *MYC*, and the PTEN/PI3K/mTOR pathway. Glutamine is also produced through a process known as macropinocytosis by stromal fibroblasts, which has been shown to be regulated by *RAS*, *PTEN*, and *PI3K*. Large EVs can also reprogram glutamine metabolism in recipient prostate cancer cells. A host of other mediators ranging from SRC-2 to CSCs have also been implicated in regulating glutamine metabolism in prostate cancer. Dashed lines represent putative relationships that remain not fully described. AR, androgen receptor; mTOR, mammalian target of rapamycin; PTEN, phosphatase and tensin homolog; PI3K, phosphoinositide 3-kinase; Gln, glutamine; EVs, extracellular vesicles; Asp, aspartate; UDP-GlcNAc, uridine diphosphate N-acetylglucosamine; Glu, glutamate; *α*-KG, *α*-ketoglutarate; TA, transaminase; GOT1, aspartate aminotransferase; NEAAs, non-essential amino acids; GSH, glutathione; NH_4_+, ammonium; GLS, glutaminase; GLUD, glutamate dehydrogenase; mal, malate; OAA, oxaloacetate; Ac-CoA, acetyl-CoA; cit, citrate; TCA, tricarboxylic acid; ME, malic enzyme; pyr, pyruvate; lac, lactate; SRC-2, steroid receptor coactivator 2; PDHA1, pyruvate dehydrogenase E1; GMPS, guanosine monophosphate synthetase; CAD, aspartate transcarbamylase and dihydroorotase; MECP2, methyl CpG-binding protein 2; DNMTs, DNA methyltransferases (DNMTs); HIF1-*α*, hypoxia-inducible factor 1-alpha; CSCs, cancer stem cells; C5a-C5aR, fifth component of complement and receptor system.

A thorough review of glutamine metabolism in cancer is beyond the scope of this review, but this relationship has been extensively reviewed elsewhere [9–12]. Glutamine is the most abundant amino acid in plasma, and although nonessential as most tissues can synthesize glutamine, glutamine becomes conditionally essential particularly for cancer cells where there is increased demand for glutamine to support periods of rapid growth and proliferation [12]. The principle manner by which glutamine supports tumor cell growth is through intermediary metabolism (Fig. 1). Here the metabolic fates of glutamine can be broadly categorized into reactions that utilize glutamine for its *γ*-nitrogen and those that use either the *α*-nitrogen or the carbon skeleton [12]. Glutamine enters the cell primarily through the SLC (solute carrier) family of transporters [13]. Upon entry into the cell, glutamine can undergo several metabolic fates. Glutamine serves as the nitrogen donor (*γ* or amide nitrogen) for *de novo* synthesis of both purine and pyrimidine nucleotides, while glutamine contributes to the synthesis of uridine diphosphate N-acetylglucosamine (UDP-GlcNAc) as part of the hexosamine biosynthesis pathway, which is important for glycosylation [9,11]. Secondary reactions that use either the *α*-nitrogen or the carbon skeleton of glutamine require conversion of glutamine to glutamate by glutaminase (GLS) enzymes [12].

There are two GLS isoforms: (1) GLS which is more broadly expressed in normal tissues but has a more established oncogenic role based on evidence that GLS is expressed in experimental tumors in animal models and (2) GLS2 which is restricted predominantly to the brain, liver, and pancreas and has more of a context dependent role in cancer [9,12]. Glutamate is a precursor to the major cellular antioxidant, glutathione, and alternatively contributes to nonessential amino acid synthesis through the action of cytosolic or mitochondrial enzymes, alanine aminotransferase (GPT) and aspartate aminotransferase (GOT1), where the *α*-nitrogen is dispersed into various pools of nonessential amino acids [11]. The carbon skeleton of glutamine also contributes to mitochondrial oxidative phosphorylation as glutamine is converted to glutamate by GLS, and the latter is converted to *α*-ketoglutarate, which enters the TCA cycle to produce other TCA cycle intermediates and eventually NADH, FADH_2_, and ATP [9,11]. To completely make use of glutamine’s carbon skeleton as a respiratory substrate, the carbon skeleton of glutamine exits from the TCA cycle as malate with subsequent conversion to pyruvate and then acetyl-CoA, the latter of which can ultimately re-enter the TCA cycle. Of note, the conversion of glutamate to *α*-ketoglutarate can occur through glutamate dehydrogenase (GLUD) in the mitochondrion or aminotransferases in the cytosol or mitochondrion [10]. Conversion through GLUD releases ammonia, while action through aminotransferases is a non-ammonia-generating process but leads to synthesis of other amino acids from glutamine, as previously described. Oxidation of glutamine also maintains redox homeostasis by reduction of NADP+ to NADPH by malic enzyme through the conversion of glutamine to pyruvate [11]. Lastly, glutamine can also serve as an alternative carbon source to fuel fatty acid synthesis through reductive carboxylation where NADPH is consumed to produce citrate [9].

Beyond intermediary metabolism, glutamine has been shown to support cellular growth and survival through involvement with multiple cell signal transduction pathways as well. For example, it has been well established that glutamine flux stimulates activation of mammalian target of rapamycin (mTOR) and extracellular signal-regulated protein kinase (ERK), which are both critical pathways that support cellular proliferation [11,14]. More recently, glutamine-induced mTORC1 activation has been shown to occur through two pathways: GLS-mediated production of *α*-ketoglutarate that facilitates translocation of mTORC1 to the lysosome downstream of Ragulator/RAG and asparagine synthetase (ASNS)-mediated generation of ATP that inhibits AMPK and completely activates mTORC1 at the lysosomal surface [15]. Glutamine has been shown to regulate protein folding, trafficking, and endoplasmic reticulum stress responses and can suppress autophagy through multiple pathways as well [9]. Glutamine plays a role in the epigenetic regulation of numerous genes, many of which are oncogenes including *MYC* and *C-JUN* [9]. Not surprisingly, transformed cells with strong KRAS, MYC, or PI3K/AKT/mTOR pathway activation have a tendency to demonstrate increased glutamine flux to sustain metabolism and survival.

## 3. Glutamine Metabolism and Prostate Cancer

### 3.1 Early Investigations in Antiglutamine Therapies

Glutamine starvation alone through glutamine-free medium has been shown to sufficiently suppress growth of prostate cancer cells *in vitro* [16–18]. It is therefore of no surprise that therapeutic strategies to target glutamine metabolism in prostate cancer have long been investigated. For example, the broadly active glutamine antagonist 6-diazo-5-oxo-L-norleucine (DON) was investigated in the 1950s as an anticancer agent across multiple malignancies including prostate cancer, but phase I-II trials were hampered by dose-limiting gastrointestinal (GI) toxicities resulting in abandonment of its clinical development [19]. Glutamine deprivation through the glutamine antimetabolite acivicin, glutamine conjugator sodium phenylacetate, the lead compound phenylbutyrate, and even early attempts to inhibit GLS were met with significant toxicities or technical challenges that have not resulted in the successful translation of these strategies into routine prostate cancer clinical care [20–24]. However, there has been a renewed interest in glutamine utilization by prostate cancer cells and its genetic regulation in prostate cancer due, in part, to a greater understanding of cancer metabolism. The advent of more selective GLS inhibitors, some of which have now made their way into early clinical development with proven safety and tolerability, has further motivated research endeavors into understanding the glutamine-prostate cancer relationship. Recent preclinical and clinical insights into the mechanisms of glutamine-driven prostate cancer progression and novel strategies to target glutamine metabolism will be the focus of the remainder of this review.

### 3.2 Androgen-Dependent Regulation of Glutamine Metabolism in Prostate Cancer

The sodium-dependent neutral amino acid transporter SLC1A5 or ASCT2 has been shown to be a key glutamine transporter in human prostate cancer cells (DU145, PC-3, LNCaP) though its expression appears to be lower in castrate-resistant cells than AR-sensitive prostate cancer cells [25,26]. Prevailing literature now suggests that ASCT2, but not ASCT1, transports glutamine although both are androgen regulated [27,28]. ASCT2 expression was significantly increased in prostate tumor samples compared to matched normal prostate tissues with levels that decreased in regressing prostate tumors but increased in castrate-resistant tumors using a LNCaP xenograft model [29]. Inhibiting glutamine uptake through inhibition of ASCT2 significantly reduced glutamine uptake, mTORC1 pathway signaling, and viability of prostate cancer cell lines, while inhibition of AR signaling only significantly reduced glutamine uptake in AR-sensistive cells but not AR-insensitive cells. These findings support a role for AR signaling regulation of ASCT2 and ASCT2-mediated glutamine transport. Androgens such as DHT significantly increased expression of ASCT2 and GLS in the AR-sensitive LNCaP cell line but not in the AR-insensitive DU-145 and PC-3 cell lines [26]. All three human prostate cancer cell lines showed decreased viability when exposed to GLS inhibition via BPTES although addition of the antiandrogen bicalutamide conferred an additive antitumor effect only to the AR-sensitive LNCaP cell line and not in AR-insensitive DU-145 and PC-3 cells. Furthermore, DHT supplementation attenutated the antitumor activity of BPTES in LNCaP cells. Of note, DU-145 cells have been regarded as a more aggressive prostate cancer cell line and are highly glutamine addicted with glutaminolysis being nearly four-fold higher than PC3 cells [30].

Gene and protein expression of GLS has also been shown to be present across AR-dependent (LNCaP, CW22Rv1) and AR-independent (DU-145, PC-3) prostate cancer cell lines with sensitivity to silencing of GLS by small interfering RNA (siRNA) in AR-independent cell lines characterized by marked tumor cell death in DU-145 and PC-3 cells, when compared to control [31]. In contrast to the expression of ASCT2, GLS expression has been shown to be significantly higher in AR-independent cell lines than AR-dependent cell lines, with the highest expression observed in DU-145 cells [26]. The downregulation of overexpressed glutamine transporters and GLS through secondary metabolic effects of AR antagonism has been shown to be AR dependent as well [32].

### 3.3 Glutamine Addiction and Evolution of Prostate Cancer

Several groups have demonstrated through metabolomics in *in vitro* and *in vivo* preclinical prostate cancer models that evolution from castration-sensitive to castration-resistant prostate cancer is characterized by increased glutamine uptake and subsequent flux through glutaminolysis, glutamine anaplerosis into the TCA cycle, and glutathione synthesis for maintaining redox balance [33,34]. Although AR signaling utilizes both glucose and glutamine to support prostate cancer cell growth and survival [35], LNCaP cells inducing expression of the AR variant AR-V7, which has classically been associated with resistance to ADT, preferentially enhance glutaminolysis as a fuel source via reductive carboxylation [35]. Development of metastases has also been associated with increased glutamine utilization as observed in the metastastic subline PC-3M, when compared to parental PC-3 cells [36]. Despite both PC-3M and PC-3 cells being AR-independent, the more aggressive and glutamine dependent PC-3M cell line demonstrated greater sensitivity to GLS inhibition than the PC-3 cell line.

In preclinical studies utilizing the LNCaP progression model where differentiation from LNCaP human prostate cancer cells to its derivatives C4, C4–2, and C4–2B displays an increasing propensity to metastasize to the bone and mirrors progression of human prostate cancer *in vivo*, C4–2B cells demonstrated an increasing reliance on glutamine for metabolism than their parental derivatives [37]. Using a similar LNCaP progression model, a separate group identified higher expression of GLS II pathway enzymes (GLS1, glutamine transaminase K or GTK, and *ω*-amidase) with progression from LNCaP to C4–2B prostate cancer cells [38]. Furthermore, large extracellular vesicles (EVs) shed by the highly bone metastatic C4–2B cells were enriched with GLS in the large EV cargo, which was not observed in parental LNCaP, C4, and C4–2 cells [37]. The progression to a bone cell-like phenotype also showed that expression of osteomimetic markers by C4–2B cells are critically dependent on glutamine metabolism. Notably, the GLS-mediated metabolic reprogramming towards increasing glutamine addiction from the androgen-dependent LNCaP to the androgen-independent C4–2B cell line was attenuated with inhibition of glutamine metabolism through the GLS inhibitor BPTES, providing a mechanism to inhibit large EV production, osteomimetic differentiation, and bone metastases.

Our group has recently characterized a role for stromal fibroblasts as a source of glutamine to support adjacent prostate cancer epithelial proliferation and therapeutic resistance [39]. We first identified that *RASAL3* (RAS protein activator-like 3), a RasGAP demonstrated to antagonize RAS signaling, was epigenetically silenced in prostatic cancer-associated fibroblasts (CAFs) resulting in RAS-mediated macropinocytosis and subsequent degradation of albumin in CAFs. The human prostate cancer epithelial cell lines CWR22Rv1 and C4–2B were not found to exhibit macropinocytosis themselves. Metabolome analysis demonstrated that CAFs generated glutamine with uptake by prostate cancer epithelia that was subsequently converted to glutamate. Expression of GLS was elevated in the prostate cancer epithelia when co-cultured with CAFs. The ensuing metabolism involved the familiar TCA cycle for the generation of ATP, validated by measuring oxygen consumption rate on a Seahorse XF. Strikingly, treatment with androgen receptor antagonists was associated with hypermethylation of the *RASAL3* promoter in CAFs and potentiated glutamine synthesis by prostatic fibroblasts that further promoted prostate cancer progression to a more aggressive and ATT-resistant state. For example, in castrated and enzalutamide treated mice, we found that tissue recombinant tumors of CAFs and CWR22Rv1 prostate cancer epithelial cells expanded despite androgen receptor and androgen synthesis inhibition (*p* < 0.0001). Treatment of co-cultures of CWR22Rv1 cells and Ras-activated mouse prostate fibroblasts with BPTES (GLS inhibitor) or GPNA (ASCT2 inhibitor) decreased epithelial cell proliferation compared to control. Treatment of tumor recombinant, castrate-resistant xenograft mouse models with GPNA also significantly decreased tumor growth compared to control. In co-cultures of CAFs with CWR22Rv1 cells, glutamine alone was able to induce neuroendocrine differentiation while knockdown of either *SLC1A5* or *GLS* by siRNA in the setting of glutamine incubation was able to reverse the effect of glutamine-induced neuroendocrine differentiation. The significance of our findings can be summarized by two important concepts: (1) prostate fibroblasts are an important source of glutamine to support adjacent prostate cancer epithelial progression and neuroendocrine differentiation and (2) fibroblastic glutamine synthesis is potentiated by hormonal therapy. Our model therefore provides a novel mechanism of prostate cancer progression to more therapeutically resistant states through stromal-epithelial glutamine signaling dependent on exposure to androgen receptor therapy.

A separate group has shown that in AR-sensitive prostate cancer cells, ADT suppresses AR function and GLS1 expression in its predominant KGA isoform, which results in a decrease in glutamine catabolism and tumor cell proliferation [40]. Eventually, prostate cancer cells regain ability to utilize glutamine as they develop resistance to hormonal therapy and progress to castration-resistance through a GLS isoform switch from KGA to the GAC isoform that is driven by *MYC*. GAC demonstrates potent enzymatic ability and facilitates increased glutamine dependency in aggressive variants of prostate cancer where GAC expression is increased such as CRPC and small-cell neuroendocrine carcinoma. In *in vitro* and *in vivo* models with the AR-independent PC-3 cell line, there was greater sensitivity to GLS1 inhibition with CB-839 owing to greater activity of GAC in PC-3 cells than the hormone-sensitive LNCaP cell line. Not surprisingly, CB-839 showed greater antitumor activity in GAC-expressing prostate cancer cell lines. Lastly, in CW22Rv1 and LNCaP cells and the TRAMP prostate cancer mouse model, nicotine use increased glutamine consumption providing support that smoking-associated prostate cancer progression may be glutamine mediated [41].

### 3.4 MYC and Other Key Regulators of Glutamine Metabolism in Prostate Cancer

Initial studies have identified that the oncogene *MYC* represents a master regulator of glutamine metabolism in prostate cancer [42,43]. In human PC-3 cells, *MYC* upregulates glutamine catabolism to produce ATP or glutathione through transcriptional repression of microRNAs miR-23a and miR-23b, which results in the increased expression of their target protein, mitochondrial GLS. Decreasing *MYC* expression by small interfering RNA (siRNA) reduced GLS expression and human PC3 prostate cancer cell proliferation accordingly. Inhibition of GLS through CB-839 similarly induced tumor death in PC-3 cells in a manner that was *MYC*-dependent [44]. Knockdown of *MYC* using short hairpin RNA (shRNA) or siRNA targeting *MYC* in LNCaP cells decreased tumor cell viability, glutamine uptake, and expression of the glutamine transporter SLC1A5 (also known as ASCT2), when compared to control, although these effects were attenuated in the presence of stable androgens [45]. Of note, *MYC* knockdown had no effect on GLS protein levels although these experiments were conducted in AR-dependent cell lines instead of the AR-independent PC-3 cell line. At the gene level, *MYC* regulation of *GLS* may be cell-line dependent as siRNA knockdown of *MYC* decreased *GLS* gene expression in LNCaP cells but not in DU-145 cells [46]. Inhbition of mTORC1 with rapamycin also suppressed the androgen-mediated expression of SLC1A5 and glutamine uptake in LNCaP and VCaP prostate cancer cells, suggesting that mTOR regulates glutamine uptake in prostate cancer [45].

The regulation of glutamine metabolism in prostate cancer by MYC may be dependent on PTEN/PI3K status as well given that MYC was unable to increase SLC1A5 expression in prostate cancer cells where PTEN and/or PI3K were wildtype [45]. In PTEN/PI3K-mutant prostate cancer, both MYC and mTOR signaling may cooperate to increase glutamine uptake and promote tumor cell growth. MYC or GLS may regulate prostate cancer cell radiosensitivity as inhibition of either increased radiosensitivity in prostate cancer cells *in vitro*, particularly in tumor cells that were glutamine dependent [46]. In glutamine-independent prostate cancer cells, inhibition of autophagy (ATG5 knockdown) reverses the pro-survival mechanism under glutamine starvation conditions via autophagy and increases radiosensitivity [46].

The Rho GTPases of the RAS superfamily, RhoA and RhoC, have shown the ability to confer sensitivity to glutamine deprivation as PC-3 cells expressing dominant negative mutations in RhoA or RhoC were more resistant to glutamine deprivation [47]. It has been suggested that Myc requires signaling mediated by Rho to reprogram glutamine metabolism in cancer cells [48]. Alternatively, the oncogenic transcriptional coregulator steroid receptor coactivator 2 (SRC-2) stimulates reductive carboxylation and reprograms glutamine metabolism in prostate cancer cells to promote tumor growth and metastasis [49]. Glutamine uptake, on the other hand, has been shown to upregulate SRC-2 activity in a mTORC1-RAG-dependent fashion to promote lipogenesis. Depletion of SRC-2 inhibited growth of C4–2 and PC3 cells in a similar manner to GLS inhibition with BPTES though at higher concentrations of BPTES, the effect on tumor cell growth was greater than that achieved with SRC-2 depletion [49].

Pyruvate dehydrogenase E1 (PDHA1) is considered the rate-limiting step for pyruvate decarboxylation and production of acetyl-CoA for entry into the TCA cycle. Its knockout in LNCaP prostate cancer cells results in increased glutamine uptake through upregulation of *GLUD1* and *GLS1*, when compared to the parental LNCaP cell line [50]. LNCaP knockouts for *PDHA1* also demonstrated increased sensitivity to GLS inhibitors BPTES and epigallocatechin-3-gallate (EGCG) compared to controls. In the *de novo* purine biosynthesis pathway, guanosine monophosphate synthetase (GMPS) utilizes glutamine to synthesize the guanine nucleotide, GMP. Inhibition or knockdown of GMPS in PC-3 and LNCaP cells inhibited tumor proliferation and delayed *de novo* nucleotide synthesis with accumulation of purine and pyrimidine nucleotides and alteration of glutamine-derived carbon usage [51]. Tumor growth in PC-3 mouse xenografts was significantly inhibited with knockdown of GMPS compared to controls reaffirming that GMPS represents a potential therapeutic target of glutamine metabolism as well.

Metabolic pathways that regulate the fate of glutamine’s carbon and nitrogen are additionally involved in regulation of glutamine metabolism in prostate cancer [52]. Firstly, the growth inhibitory effects of glutamine starvation in human AR-independent prostate cancer cell lines can be rescured with addition of *α*-ketoglutarate, non-essential amino acids, and nucleosides, underscoring the importance of both glutamine carbon and nitrogen for prostate cancer survival. In AR-independent PC3 and C4–2MDVR cells, there is greater assimilation of glutamine amine nitrogen for pyrimidine nucleotide synthesis than AR-positive LNCaP and C4–2 cells, while glutamine’s carbon appears not to be a major contributor to purine synthesis in prostate cancer. Interestingly, aspartate transcarbamylase and dihydroorotase (CAD), an enzyme involved in pyrimidine synthesis found to be upregulated in advanced CRPC and small-cell neuroendocrine carcinoma, is involved in the reciprocal regulation of glutamine metabolism. Knockout of CAD in prostate cancer cells results in increased glutaminolysis, while suppressing GLS1 results in elevated pyrimidine synthesis. This reciprocal regulation of glutamine carbon and nitrogen catabolism was abolished when both CAD and GLS1 were suppressed, suggesting that blocking both pathways maximally inhibits glutamine utilization by prostate cancer cells [52].

Glutamine metabolism in prostate cancer cells has also been shown to be regulated by methyl CpG-binding protein 2 (MeCP2) and DNA methyltransferases (DNMTs) that cooperate to promote active methylation of the miR-137 promoter, resulting in decreased miR-137 transcription and enhanced TRIM24 expression [53]. Regulation of glutamine trafficking is not surprisingly dependent on mitochondrial integrity as well given that treatment of prostate cancer cells with monoethanolamine (Etn) resulted in cellular lipid accumulation, alteration of mitochondrial structure, and induction of lipid-mediated activation of cell death pathways [54]. Treatment of PC-3 *in vitro* and *in vivo* models with Etn additionally downregulated HIF1-*α* function, downregulated enzymes of glutamine metabolism, decreased intracellular glutamine levels, and activated p53-induced cell death. Depletion of inctracellular levels of glutamine with Flavokawain A, a kava root extract, in PC3 cells also resulted in tumor cell death thought to be due to decreased glutathione synthesis and generation of reactive oxygen series (ROS) [55].

In experiments with the AR-independent human PC-3 cell line, dependency on glutamine for mitochondrial respiration appeared to be related to the presence of metastatic subpopulations enriched in cancer stem cell (CSC) features where these PC-3-derived cells (PC-3M) showed that glutamine signaling through the TCA cycle was more pronounced than in mesenchymal-like non-CSC PC-3-derived cells (PC-3S) [56]. Reductive carboxylation and higher GLS1 expression was observed in PC-3M cells than PC-3S cells with a greater inhibition of proliferation in PC-3M cells to the GLS inhibitor BPTES. Growth inhibition of PC-3M cells by BPTES was notably attenuated with Snai1 overexpression. Glutamine metabolism has also shown to mediate epigenetic reprogramming and regulation of CSC populations in prostate cancer *in vivo* mouse models [46]. This regulation appears to be reciprocal given that inhibition of histone methylation by the epigenetic CSC inhibitor DZNeP downregulates GLS expression.

Finally, the complement system was recently implicated in the regulation of glutamine metabolism in castrate-resistant prostate cancer (CRPC) [57]. Glutamine consumption in human prostate cancer PC-3 cells was increased in a C5a concentration-dependent manner. Use of a C5a antagonist, which is a byproduct from the fifth component (C5) of complement, attenuated glutamine consumption in PC-3 cells only in the presence of C5a. Consumption of glutamine in CRPC, therefore, may be reulgated in part by the C5a-C5aR system. Table 1 (Ref. [26,29,31,36,37,39,40,42,45,46,49–52,54–56,58]) summarizes the sensitivity of prostate cancer cells from key early preclinical investigations of antiglutamine therapies.

### 3.5 Glutamine as a Biomarker in Patients with Prostate Cancer

Given the growing evidence that prostate cancer progression is characterized by an increasingly glutamine dependent state, it is unsurprising that there have been large efforts into exploring the use of glutamine metabolism as a marker of prostate cancer aggressiveness and therapy resistance in patients. A potential biomarker role for glutamine in prostate cancer was demonstrated in human prostate adenocarcinoma tissue microarrays whereby tumor expression of the glutamine transporter ASCT2 was correlated with more aggressive biological behavior [59].

In an early series, serum glutamate levels were significantly increased in men with primary prostate cancer but returned to normal in men with mCRPC. Among primary prostate cancer cases, serum glutamate levels were significantly associated with Gleason score and African-American race [60]. Our group, however, has shown that glutamine loses its prognostic value in those with localized prostate cancer where plasma glutamine was measured at the time of radical prostatectomy [61]. A similar finding was observed with tissue GLS1 expression where its prognostive value was lost in a predominantly localized prostate cancer cohort [62]. Our findings are consistent with data that it is not until advanced stages with sustained exposure to ADT when prostate cancer becomes more addicted to glutamine along its clinical course. In advanced prostate cancer patients treated with ADT, we have shown that plasma glutamine is prognostic [39]. In a cohort of patients with mCRPC, plasma glutamine levels were predictive and correlated negatively with prostate specific antigen doubling time (PSA-DT) [46]. Fasting plasma levels of glutamine has been associated with an increased risk of developing prostate cancer during 13 years of follow up [63].

On tissue metabolomic analyses of patients having prostate cancer recurrence, glutamine and glutamate were among the major metabolites contributing to a profile associated with tumor recurrence [64]. Tissue glutamate levels have been shown to be significantly increased with disease progression from benign to prostate cancer to metastatic prostate cancer [49]. This is consistent with another group where among the major tissue metabolites measured in metabolomic profiling, glutamine levels were shown to increase from early-stage prostate cancer to CRPC [65].

Metabolomic profiling in prostate tumors and matched adjacent normal tissues have shown that multiple mediators involved with glutamine catabolism such as intermediates of the TCA cycle were elevated in prostate cancer samples, offering putative mechanisms of prostate cancer pathophysiology [66]. Tumor GLS1 expression was highly correlated with tumor stage and progression in prostate cancer patients compared to benign prostatic hyperplasia tissues [67]. In another study, GLS1, *ω*-amidase, and GTK (all enzymes in the GLS pathway) had negligible expression in the stromal cell compartment of the normal prostate, but were expressed in the stroma of cancerous human prostate tissues with higher staining intensity associated with increasing Gleason grade [38]. In prostate cancer samples from a separate cohort, higher protein expression of GLUD1 and GLS1 correlated with Gleason scores and poorer overall survival, again suggesting a potential for key enzymes of glutamine metabolism to serve as prognostic biomarkers for prostate cancer [50]. GMPS mRNA expression additionally correlated with Gleason score in prostate cancer samples, while high GMPS expression was associated with metastatic disease, higher grade, and decreased rates of overall and disease/progression-free survival [51].

Beyond blood and tissue, urine glutamine levels were highest in prostate cancer compared to other genitourinary cancers bladder and renal cell carcinoma [68]. Lastly, given the unique reliance for prostate cancer on glutamine for survival and proliferation, there is a growing effort to develop glutamine as a novel tracer for innovative imaging of prostate cancer and other malignancies. This topic has been extensively reviewed and is beyond the scope of this review [69,70].

## 4. Therapeutic Targeting of Glutamine Metabolism: Future Considerations for Translation to the Clinic

The first-in-human, open-label, phase I trial of telaglenastat (CB-839), a first-in-class, small molecule, oral allosteric and selective inhibitor of GLS, recently reported final results in patients with treatment-refractory or advanced solid tumors [71]. Unlike its predescessors, telaglenastat demonstrated exceptional tolerability (fatigue (23%) and nausea (19%) being the most common adverse events) with the majority of toxicities being grade 1–2. A maximum tolerated dose was not reached, but 800 mg twice-daily of telaglenastat was the recommended phase II dose based on favorable pharmacokinetic and pharmacodynamic profiles. With the promising safety and tolerability of telaglenastat demonstrated in phase I trials, it is of no surprise that clinical development is expanding for therapeutic agents that target glutamine metabolism across a multitude of malignances. However, there are several key considerations that must be accounted for in order to (1) optimize the efficacy of inhibiting glutamine metabolism and (2) successfully translate clinical-grade inhibitors to patients with prostate cancer.

### 4.1 Adaptive Resistance to Blocking Glutamine Metabolism through Compensatory Metabolic Pathways

Early experiences with GLS inhibition have demonstrated that glutamine metabolism in tumors is complex with cancer cells having the ability to adapt and alter metabolic pathways that promote resistance to monotherapy GLS inhibition [19]. Metabolic adaptations have been demonstrated in prostate cancer cell lines (DU-145 and PC3) as well to glutamine restriction [72]. Here, acute glutamine deprivation increased glucose consumption, decreased lactate production, elevated the NAD/NADH ratio, increased mitochondrial glutathione peroxidase activity, and increased mitochondrial pyruvate dehydrogenase (PDH) activity and accumulation of ROS levels. In multicellular tumor spheroids derived from the AR-independent DU-145 human prostate cancer cell line, incubation of tumor spheroids in glutamine-reduced cell culture medium stimulated prostate cancer spheroid growth in a manner dependent on intracellular ROS elevation and regulation of mitogen-activated kinase (MAPK) signaling and the multidrug resistance (MDR) transporter P-glycoprotein (Pgp) [73].

Furthermore, although inhibition of key enzymes of glutamine nitrogen and carbon catabolism (CAD or GLS1, respectively) suppressed growth of prostate cancer cell lines, this inhibitory effect diminished over time suggesting that tumor cells acquire resistance through compensatory mechanisms after either pathway is inhibited [52]. For example, loss of CAD enhanced glutaminolysis-related and ammonia assimilation pathways, while GLS1 inhibition enhanced pyrimidine synthesis activity. In *PTEN*-deficient PC-3 and C4–2MDVR cells, upregulation of CAD was mediated by the PI3K-AKT-mTOR-S6K signaling axis. Interestingly, in transgenic mouse models with variable expression of *PTEN*, PTEN negatively regulates GLS through degradation by the E3 ubiquitin ligase anaphase-promoting complex/cyclosome-Cdh1 (APC/C-Cdh1) [74]. As *PTEN* loss is highly prevalent in prostate cancer, consideration of *PTEN* status when employing GLS inhibition as a therapeutic strategy will be pivotal and further investigation is warranted into this unique relationship.

Adaptive resistance mechanisms to GLS1 inhibition extends beyond upregulation of mitochondrial oxidative phosphorylation as well. Growth suppression of human prostate cancer cell lines via BPTES, for example, was not rescued with supplementation of cell-permeable *α*-ketoglutarate despite knowledge that glutamine is an anaplerotic TCA cycle substrate [56]. Instead, glutamine likely exerts a survival function independent of its anaplerotic function in prostate cancer cells whereby epithelial and pluripotency gene programs have been shown to confer sensitivity to glutaminase inhibition dependent on presence of specific CSC subpopulations [56].

### 4.2 The Role of the Tumor Microenvironment in Glutamine Signaling and Prostate Cancer Progression

The large majority of preclinical studies has focused on targeting glutamine uptake and utilization in the prostate cancer epithelia as a therapeutic approach. However, as our group has demonstrated, the tumor microenvironment plays an important role in facilitating glutamine synthesis for utilization by adjacent prostate cancer epithelial cells to support their growth and evolution [39]. Specifically, RAS-mediated macropinocytosis and subsequent degradation of albumin in stromal fibroblasts facilitates production of glutamine to support adjacent prostate cancer epithelial proliferation. We also showed that RAS-mediated fibroblastic glutamine synthesis is potentiated by exposure to androgen pathway inhibition thereby providing a mechanism for prostate cancer progression through the course of treatment with hormonal therapy, a foundational treatment in prostate cancer. Notably, we observed that the human prostate cancer epithelial cell lines CWR22Rv1 and C4–2B were not found to exhibit macropinocytosis themselves. A separate group has demonstrated that *PTEN* loss and PI3K pathway activation in fibroblasts promotes macropinocytosis, while select human prostate cancer cell lines with *PTEN* deletion may also exhibit macropinocytosis in an AMPK-dependent manner [75].

In co-cultures of stromal fibroblasts and prostate cancer cells, p62-deficiency in the stroma was able to sustain prostate cancer proliferation despite glutamine deprivation, offering insight into a novel mechanism whereby stromal cells and epithelial prostate cancer cells are rendered resistant to glutamine deprivation [76]. Here, stromal p62 deficiency leads to ATF4 upregulation and metabolic reprogramming to stimulate glucose flux through a pyruvate carboxylase-asparagine synthase cascade that ultimately results in asparagine production as a source of nitrogen for stromal and prostate cancer epithelial proliferation. Interestingly, in ovarian cancer preclinical studies the enzyme glutamine synthetase (GS or GLUL), which catalyzes condensation of glutamate and ammonia to synthesize glutamine, showed a higher expression in ovarian CAFs compared to normal ovarian fibroblasts as well as higher expression in the stromal component than epithelial component of ovarian cancer patient-derived tumor tissues [77]. Targeting GLUL in the stroma and GLS in cancer cells inhibited tumor proliferation and metastasis in an ovarian carcinoma orthotopic mouse model.

Pertaining to the prostate cancer epithelial expression of GS/GLUL, it should be noted that one group has shown that mRNA and protein expression of this enzyme is present in androgen-independent PC-3 and androgendependent LNCaP prostate cancer cells [78]. This is contradictory to another group whereby mRNA expression for *GLUL*, the gene encoding glutamine synthetase, was undetectable in the androgen-sensitive LNCaP and VCaP cell lines with or without treatment with exogenous androgens [45]. The role of GLUL in supporting glutamine-driven prostate cancer growth in both stromal and epithelial components remains poorly described.

A large class of EVs (1–10*μ*m diameter) known as large oncosomes (LO) have been shown to be shed as byproducts of non-apoptotic plasma membrane blebbing from prostate cancer cells [79]. Among the proteins enriched in large EVs include GLS and those involved in glutamine metabolism. Remarkably, exposure to large EVs altered glutamine metabolism in recipient prostate cancer cells (DU145) with an increase in GOT1 expression, an aminotransferase that converts aspartate and *α-*ketoglutarate to glutamate, within 2 hours of exposure indicative of protein and not mRNA transfer via large EVs to recipient prostate cancer cells. EVs therefore represent another component of the tumor microenvironment that can potentially regulate glutamine metabolism and prostate cancer progression. In short, the constellation of these novel findings underscore the need to consider both the tumor stroma and epithelial components in targeting glutamine metabolism and for understanding resistance pathways to glutamine deprivation strategies in prostate cancer.

### 4.3 Glutamine as a Resistance Mechanism to Prostate Cancer Therapies

Glutamine has been implicated in contributing to resistance with standard therapies in prostate cancer as well. In docetaxel-resistant PC-3 cells (PC3-DR) established by increasing doses of docetaxel in sensitive PC-3 cells, a pro-invasive phenotype characterized by epithelial-to-mesenchymal-transition (EMT) markers and a decrease in intracellular ROS [80]. Moreover, the metabolism of PC3-DR cells shifts towards increased glutamine uptake in the setting of *MYC* upregulation and mitochondrial oxidative phosphorylation to sustain the acquisition of the docetaxel resistance phenotype. Docetaxel resistance was also shown to be contributed by CAFs through co-cultures with taxane-sensitive PC-3 cells that conferred resistance. In mCRPC patients receiving treatment with docetaxel or cabazitaxel having blood principally collected pretreatment, those who received treatment with abiraterone or enzalutamide prior to taxanes had significantly higher plasma glutamine levels than those who did not receive those therapies prior to the taxanes [81]. Lower glutamine levels were associated with response to taxanes, while high glutamine levels were associated with shorter time to progression and overall survival.

With respect to radiation therapy, prostate cancer patients having high tumor gene expression of *GLS1* and *MYC*, key regulators of glutamine metabolism, were shown to have significantly decreased progression-free survival with radiation therapy [46]. In DU-145 prostate cancer cells, it was shown that glutamine starvation cells resulted in significant radiosensitization, while a combination of glutamine depletion and metformin treatment significantly increased radiosensitizing effects. In a cohort of prostate cancer patients having received prostate biopsies prior to radiation therapy, glutamine was a potential prognostic indicator of recurrence following neoadjuvant hormonal therapy and radiation therapy [82]. As such, there is growing evidence to suggest that inhibiting glutamine metabolism may also confer sensitivity to standard prostate cancer therapies in addition to offering cancer therapeutic effects itself.

### 4.4 Novel Approaches to Target Glutamine Metabolism and Strategies to Exploit Synergism

As described previously, evidence is mounting to suggest that prostate cancer is highly adaptive with compensatory metabolic networks that inevitably facilitate resistance to glutamine blockade, particularly with monotherapy. It is therefore likely that future efforts to target glutamine metabolism will need to elicit combinatorial strategies that address adaptive metabolic and related signaling cascades to enhance sensitivity to glutamine blockade. Combination strategies are increasingly feasible given the promising safety and tolerability of telaglenastat (CB-839), an oral selective inhibitor of GLS that renders it an attractive agent for combination with other cancer therapeutics. Indeed, multiple groups have now generated preclinical data to support the rational combination of various therapeutic strategies to increase sensivity to glutamine metabolism inhibition.

Glutamine deprivation has previously been shown to induce DNA damage response as measured by upregulation of the DNA damage response marker *γ*H2A.X in AR-dependent and AR-independent prostate cancer cell lines while radiosensitizing cells to radiation therapy [46]. *In vitro* data has supported that CB-839 in combination with poly (ADP-ribose) polymerase (PARP) inhibitors niraparib and talazoparib led to synergistic anti-proliferative activity in prostate cancer cells and other solid tumor cell lines [83]. Telaglenastat is being explored in combination with talazoparib in a phase II clinical trial of patients with treatment-refractory, DNA damage repair mutation negative mCRPC (NCT04824937).

Under hypoxic conditions, hypoxia-inducible factor-1*α* (HIF-1*α*) is elevated and in the setting of loss of von Hippel-Lindau protein that targets HIF-1*α* for proteasomal degradation, HIF-1*α* stabilizes and is consitutively expressed resulting in upregulation of hypoxia-responsive target genes involved in cell survival, angiogenesis, and metabolism. In DU-145 cells, glutamine deprivation resulted in more effective inhibition of HIF-1*α* protein levels when compared to glucose deprivation [84]. In human prostate cancer cell lines (PC-3 and LNCaP), inhibition of HIF-1*α* rescues inhibitor of DNA binding 1 (ID1) from degradation and ultimately results in upregulation of *GLS2* and glutamine metabolism, switching the metabolic dependency of HIF1*α*-inhibited cells from glucose to glutamine [85]. These preclinical findings suggest a rationale to marry HIF-1*α* and glutamine uptake inhibition as a therapeutic strategy warranting further investigation in prostate cancer.

Targeting multiple cascades of glutamine signaling has proven efficacy in preclinical prostate cancer models as well. Using patient-derived xeonografts of CRPC, dual mTORC1/2 inhibition with RapaLink-1 reduced prostate cancer bone metastasis viability and effectively diminished metabolic enzymes that are linked to glutamine metabolism such as GFAT1, GS, GLS, and CAD [86]. In the setting of glutamine deprivation, vitamin C induced a cytotoxic effect on PC-3 prostate cancer cells that was correlated with GS/GLUL expression [87]. Vitamin C treatment was associated with increased ROS levels, depletion of glutathione, and NADPH/NADP+ reduction. Pharmacologic vitamin C treatment in prostate cancer xenografts overexpressing GS showed more significant therapeutic effects, reinforcing that vitamin C could elicit cytotoxic effects in cancer cells by targeting GS and inducing oxidative stress. In anoxia-tolerant cells characterized by upregulated GOT1 expression, inhibition of glutamine-dependent glutathione metabolism has been shown to provide a means to overcome resistance induced by chronic hypoxia and ROS defense [88].

Glutamine feeds into the hexosamine biosynthesis pathway through intermediary metabolism, whereby in PC-3 cells UDP-GlcNAc declined with glucose starvation but increased with glutamine abundance [89]. GlcNAc supplementation subsequently increased glucose and glutamine uptake and catabolism in PC-3 cells. Inhibition of the hexosamine biosynthesis pathway, therefore, in tumors with PC-3-like metabolism may serve to suppress metabolism and down-regulate cell growth, which is heavily dependent on glutamine. UDP-GlcNAc is an obligatory substrate for O-linked *β*-N-acetylglucosamine (O-GlcNAc) transferase (OGT), and OGT has been shown to have higher expression in prostate cancer compared to normal prostate [90]. Targeting OGT reduced eexpression of matrix metalloproteinase (MMP)-2, MMP-9, and VEGF, thereby suppressing invasion and angiogenesis in prostate cancer cells. In a prostate cancer mouse model, blocking OGT expression inhibited bone metastasis and provided further support that OGT represents a novel target of glutamine intermediary metabolism [90].

Glutaminolysis has also been known to generate ammonia (Fig. 1). In growing tumors that are therefore glutamine addicted, ammonia release can be used by surrounding tumor cells to increase autophagy and removal of toxic by-products as a protective mechanism for tumor cells [91,92]. Interestingly, in non-prostate cancer preclinical models, overexpression of sirtuin 5 (SIRT5, a class III histone deacetylase) has been shown to reduce ammonia accumulation whereas SIRT5 inhibition increases GLS expression and leads to increased ammonia accumulation and autophagy induction [93]. SIRT5 activation therefore represents a possible avenue to regulate glutamine metabolism, reduce ammonia-induced autophagy, and impair the ability for cancer cells to survive under stress, although this remains untested in prostate cancer. Given the intricate relationship between glutamine metabolism and autophagy, dual targeting of glutamine metabolism and autophagic pathways such as mitogen-activated protein kinase (MAPK) and PI3K/AKT/mTOR may represent a promising approach to treat therapy-resistant prostate cancer [94,95]. Autophagic-modulatory drugs are currently being developed in clinical trials to regulate autophagy and mitigate therapy resistance to anticancer therapies [95].

Our group has shown that neuroendocrine differentiation is inducible with glutamine in CWR22Rv1 cells, while inhibition of mTOR with rapamycin restoring expression of neuroendocrine genes to control levels [39]. Given that glutamine activates mTOR leading to neuroendocrine differentiation, combined mTOR and glutamine signaling inhibition remains a logical but untested strategy in the clinical setting. Upstream to mTOR, other groups have shown that glutamine deprivation reduced the GTP binding activity of Rho protein and Ras-GTP levels in AR-independent prostate cancer cells [18]. Glutamine restriction in PC-3 cells has shown to reduce Akt activity as well [96]. In sum, glutamine deprivation may represent a strategy to enhance suppression of cell survival signaling cascades including mediators of the MAPK and mTOR/Akt pathways.

Combining glucose and glutamine metabolism inhibition has also demonstrated early signs of feasibility in prostate cancer. In LNCaP, PC-3, and DU-145 human prostate cancer cells, decreasing glutamine flux via GLS inhibitors (compound 968 or BPTES) increased sensitivity to metformin [58]. Metformin can upregulate glutamine anaplerosis in LNCaP cells, but addition of BI2536, a polo-like kinase 1 (Plk1) inhibitor, antagonizes this effect and ultimately results in prostate cancer cell death due to energy crisis [97]. A separate study has shown that memantine, which has demonstrated glutamate metabolism-interfering properties, paired metformin inhibited MYC expression and decreased HIF-1*α* levels while eliciting antineoplastic effects in the AR-dependent LNCaP prostate cancer cell line [98]. Alkalinization of extracellular pH has also shown to alter cellular metabolism to a nutrient consumption-dependent state susceptible to glucose and glutamine deprivation in neuroendocrine prostate cancer cell line [99].

Paradoxically, other groups have shown that derivatives of glutamine and glutamic acid induced growth inhibitory activity in PC-3 cell lines, with some derivatives showing cytotoxicity comparable to chemotherapy [100]. The antineoplastic effects of glutamine supplementation appears context-dependent in prostate cancer as addition of glutamine into starvation media restored uniform distribution of valosin-containing protein with ATPase activity (VCP) to unexpectedly induce rapid prostate cancer cell death that was characterized by ferroptosis of VCP. Notably, necrotic cell death occurred only with glutamine supplementation in PC-3 cells cultured in amino acid-deprived conditions, while this phenomenon did not occur with supplementation of other individual amino acids [101]. Despite these unexpected findings that warrant further validation, the overwhelming majority of the field continues to explore drug development in combinations with agents that inhibit glutamine metabolism. For example, high-throughput screening of natural compounds has identified that multiple combinations of natural compounds that resulted in synergistic antitumor activity in preclinical prostate cancer models possess mechanisms that modulate glutamine metabolism such as blockade of glutamine uptake [102].

## 5. Conclusions

A multitude of preclinical studies have suggested that glutamine addiction is a hallmark of prostate cancer progression. Glutamine metabolism canonically supports cellular survival and proliferation through intermediary pathways that lead to: (1) *de novo* synthesis of both purine and pyrimidine nucleotides and UDP-GlcNAc as part of the hexosamine biosynthesis pathway, (2) fatty acid synthesis through reductive carboxylation, (3) maintenance of redox homeostasis, and (4) GSH synthesis, production of NEAAs, and mitochondrial oxidative phosphorylation when glutamine is converted to glutamate. Here, GLS is a key enzyme that converts glutamine to glutamate where the latter is converted to *α*-KG for entry into the TCA cycle to produce other intermediates and eventually NADH, FADH2, and ATP. The regulation of glutamine metabolism in prostate cancer has been shown to involve the AR pathway, *MYC*, and the PTEN/PI3K/mTOR pathway. The tumor microenvironment also plays a role in facilitating glutamine signaling to support prostate cancer growth through macropinocytosis by stromal fibroblasts and production of large EVs that reprogram glutamine metabolism in recipient prostate cancer cells. These pathways represent prime targets for therapeutic development to target the glutamine dependency of prostate cancer.

## Figures and Tables

**Table 1.** Sensitivity of prostate cancer cell lines to therapeutic targeting of glutamine metabolism.

Human prostate cancer cell line	AR status	Sensitivity to inhibition of glutamine metabolism	Ref.
PC-3	Independent	Sensitive to RNA interference (siGLS, sh18, sh19, shASCT2, shGAC, and shGMPS-41/42), decoyinine, compound 968, doxycycline-inducible knockdown of GLS1, CB-839, BenSer, GPNA, Etn, FKA, and BPTES Resistant to shKGA	[26,29,40,42,49,51,52,54,55,58]
PC-3 derived cells	Independent	Sensitive to BPTES and CB-839	[36,56]
DU-145	Independent	Sensitive to RNA interference (siGLS), decoyinine, compound 968, BenSer, Etn, and BPTES Resistant to CB-839	[26,31,46,51,54,58]
C4–2	Independent	Sensitive to RNA interference (sh19) and BPTES	[49]
C4–2B	Independent	Sensitive to BPTES and Etn	[37,54]
C4–2MDVR	Independent	Sensitive to RNA interference (shGLS1), doxycycline-inducible knockdown of GLS1, and CB-839	[52]
CWR22Rv1	Independent	Sensitive to BPTES, CB-839, and GPNA	[39,46]
LNCaP	Dependent	Sensitive to RNA interference (shASCT2, shSLC1A5, shGAC, shKGA, and shGMPS-41/42), decoyinine, compound 968, BenSer, GPNA, ECGG, and BPTES Resistant to CB-839 and siGLS1	[26,29,40,45,46,50,51,58]
VCaP	Dependent	Resistant to RNA interference (siSLC1A5)	[45]

siGLS, small interfering RNA for GLS; sh18 and 19, short hairpin RNA for SRC-2; shASCT2, short hairpin RNA for ASCT2; shGAC, short hairpin RNA for GAC; shGMPS-41/42, short hairpin RNA for GMPS-41 or −42; decoyinine, GMPS inhibitor; compound 968, GLS1 inhibitor; BenSer, ASCT2 inhibitor; GPNA, ASCT2 inhibitor; Etn, monoethanolamine; FKA, Flavokawain A; BPTES, GLS1 inhibitor; shKGA, short hairpin RNA for KGA; shSLC1A5, short hairpin RNA for SLC1A5 (also known as ASCT2); ECGG, epigallocatechin-3-gallate (GLS1 inhibitor); siSLC1A5, small interfering RNA for SLC1A45 (also known as ASCT2).
